# International Dental Congress, Paris, France, August 8–11, 1900

**Published:** 1899-11

**Authors:** W. E. Griswold

**Affiliations:** 423 Mack Block, Denver, Colo.


### NOTICES.

### International Dental Congress, Paris, France, August 8-14, 1900.

The Committee appointed by the National Dental Association at the Omaha meeting August 30, 1898, was, by order of the Chairman, Dr. A. W. Harlan, convened at the Cataract House, Niagara Falls, August 1, 1899. No quorum being present, was adjourned to the 3d inst., at 4 P. M.

There were then present A. W. Harlan, of Chicago; H. A. Smith, Cincinnati; Thomas Fillebrown, Boston; T. E. Weeks, Minneapolis; J. D. Patterson, Kansas City; H. W. Morgan, Nashville; T. W. Brophy, Chicago; W. C. Barrett, Buffalo; W. W. Walker, New York City; W. E. Griswold, Denver, Colo.; B. Holly Smith, Baltimore; J. Taft, Cincinnati, Ohio.

The meeting was called to order by the chairman who gave a short address, stating the object, organization, etc., of the Congress, and the work necessary for the committee to accomplish in this country.

On motion of Dr. Weeks, W. E. Griswold was elected Secretary.

On motion of Dr. Fillebrown, Dr. Wm. Jarvie, of Brooklyn, N. Y., was elected an additional member of the committee.

On motion of Dr. Smith, the Chairman and Secretary were instructed to confer with the National Association in regard to arranging an earlier meeting next year to accommodate those going abroad.

On motion of Dr. Weeks, H. S. Sutphen, of Newark, N. J., was made a member of this committee.

On motion of Dr. Barrett, a place on this committee was reserved for the President of the National Association in the year 1900, and the chairman was authorized to insert his name.

On motion of Dr. Brophy, George H. Chance, of Portland, Oregon, was made a member of this committee.

On motion of Dr. Barrett, a resolution requesting any member of this committee finding himself unable to go abroad to attend this Congress shall at once resign, and that the executive committee be empowered to fill the vacancy, was passed.

On motion of Dr. Smith, the chairman was requested to appoint a transportation committee composed of Dr. Jarvie, Dr. Walker, Dr. Harlan and Dr. Griswold.

On motion, a committee consisting of Dr. Brophy, Dr. Weeks, Dr. Morgan, was appointed to take charge of the exhibit of American educational methods.

On motion, an executive committee of five was appointed, consisting of Dr. A. W. Harlan, Chairman, Dr. Barrett, Dr. Brophy, Dr. E. C. Kirk and Dr. H. A. Smith.

On motion, a transportation committee was appointed, consisting of Dr. Jarvie, Dr. Walker, Dr. Harlan and Dr. Griswold.

On motion, the executive committee was empowered to fill vacancies in this committee.

On motion, adjourned, to meet at call of chairman.

W. E. Griswold, *Sec'y*,
423 Mack Block, Denver, Colo.

